# Supplementary material for: Systematic dissection of key factors governing recombination outcomes by GCE-SCRaMbLE
Source: Nat Commun. 2022 Oct 3;13:5836. doi: 10.1038/s41467-022-33606-0 (PMC9530153; doi:10.1038/s41467-022-33606-0)
Supplement: Supplementary file 1 — Supplementary Information file [file 41467_2022_33606_MOESM1_ESM.pdf]

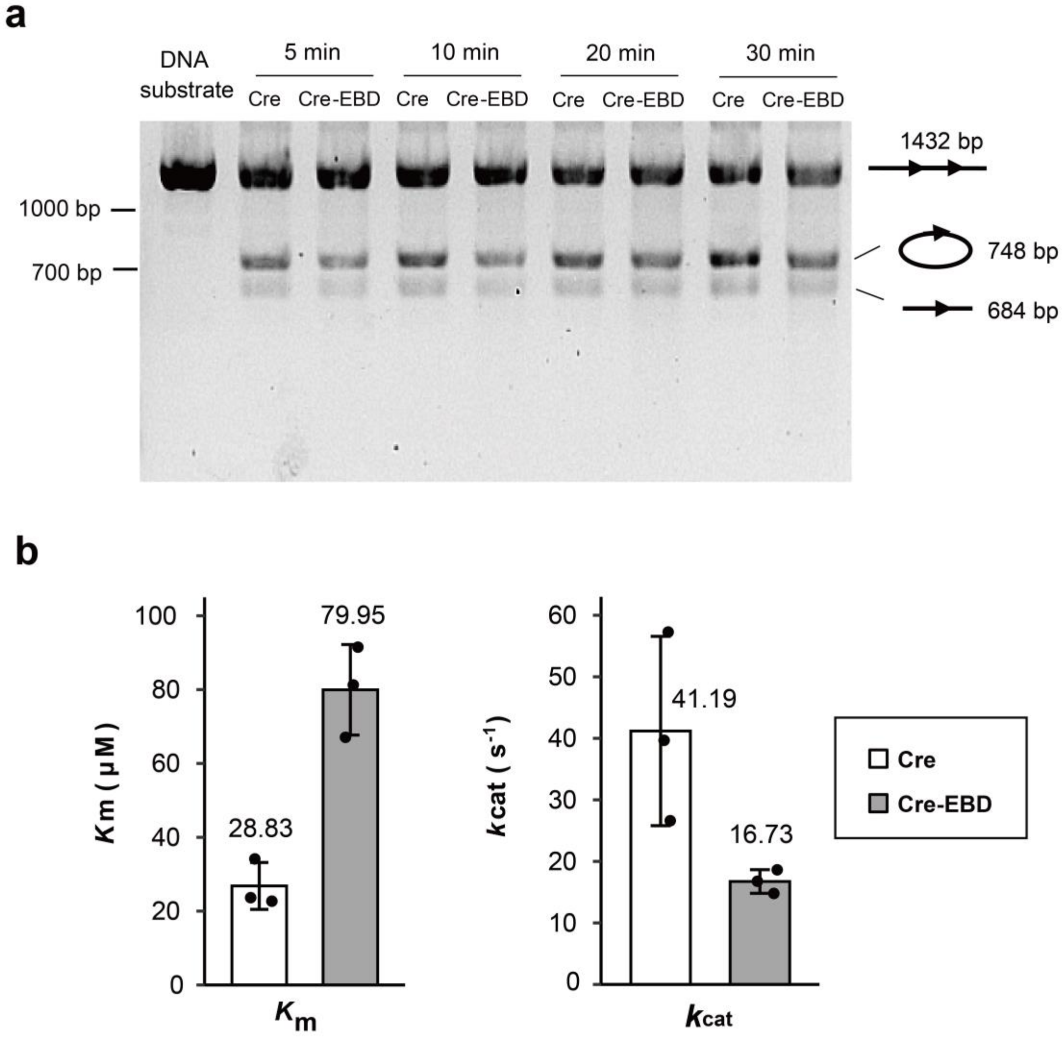

Supplementary Figure 1. *In vitro* recombination assay of purified Cre and Cre-EBD enzymes. **a** A 1432 bp linear DNA substrate containing direct repeats of the *loxP* site (triangles) is incubated with purified Cre and Cre-EBD recombinase under different time. Catalyzation of DNA substrate by Cre enzyme results in production of a 748 bp circular product and a 684 bp linear product through intra-molecular excision. The quantity of DNA substrate and product were analyzed by agarose gel electrophoresis. Experiments were performed in triplicate, and representative images are shown. **b** Comparison of *K*m and *k*cat between the Cre (white) and Cre-EBD (gray) enzymes; Data represent mean results ± s.d. from three independent experiments. Source data are provided as a Source Data file.

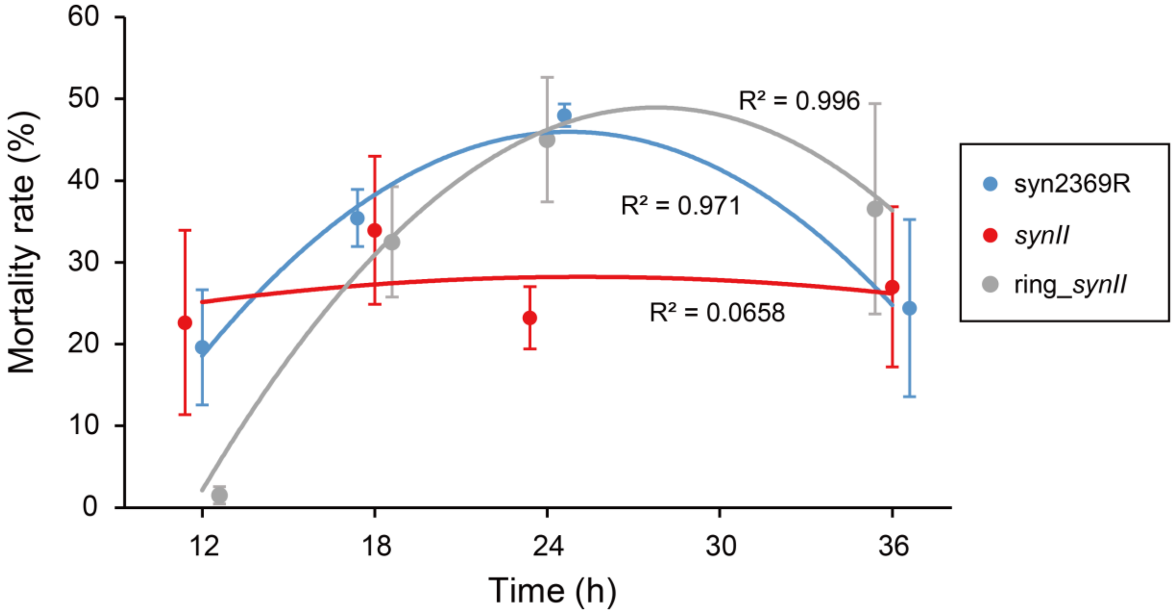

Supplementary Figure 2. Investigating the mortality rate of SCRaMbLEd synthetic yeast cells. The cell culture of haploid synthetic yeast strains harboring syn2369R (*synII*, *synIII*, *synVI* and *synIXR*) (blue), *synII* (red) and ring_*synII* (gray) undergoing GCE-SCRaMbLE in the presence of 1 mM OmeY were plated on synthetic complete (SC) medium at different timepoint. To better show the data points for three different groups at the same time points, the data points are slightly shifted from their original coordinates on X-axis. The survival rate is calculated by dividing the number of viable colonies by total colonies undergoing GCE-SCRaMbLE. The total colonies were normalized based on the unSCRaMbLEd group. Mortality rate is equal to 100% minus survival rate. Lines are second-degree polynomial curves of best fit, with R^2 values stated. Source data are provided as a Source Data file.

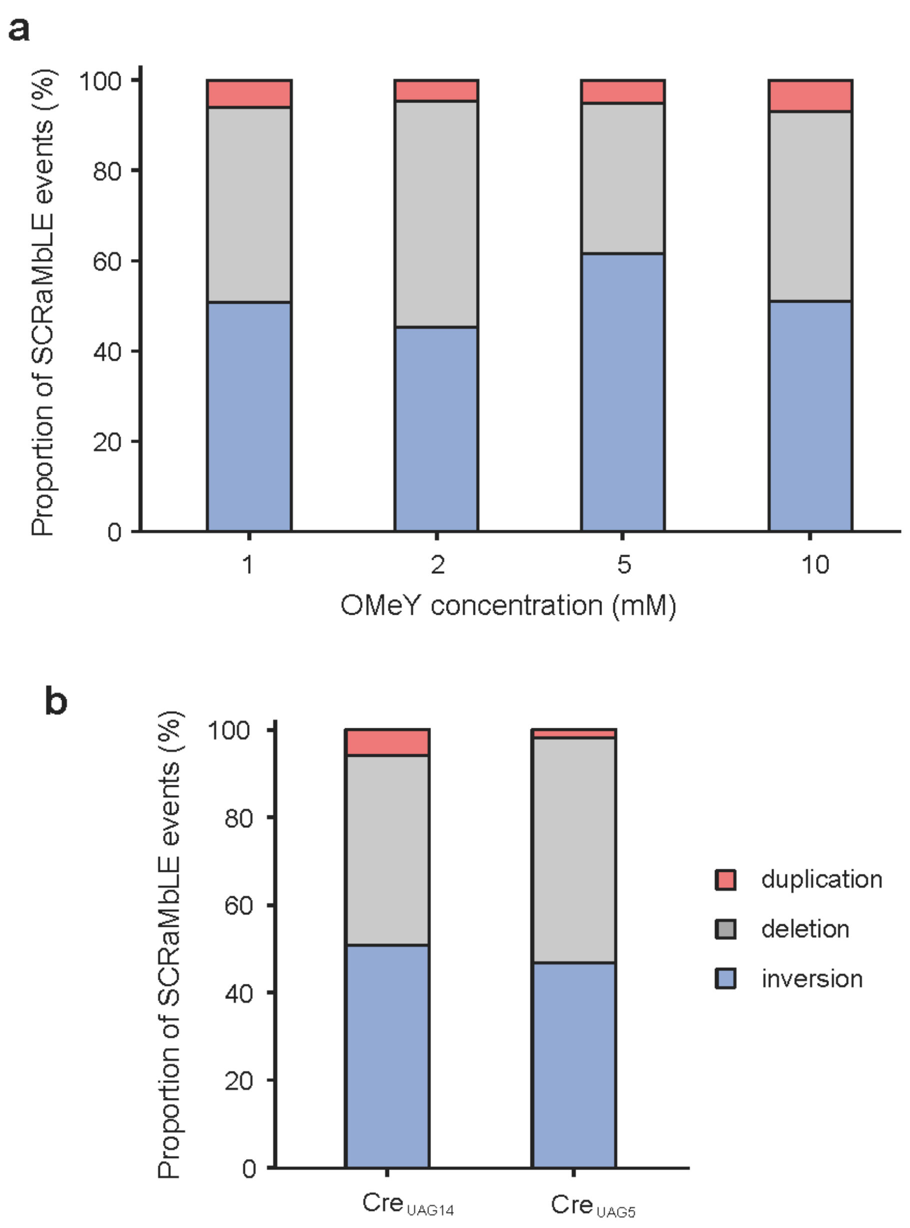

Supplementary Figure 3. The proportion of each type of recombination events including inversion (blue), deletion (gray) and duplication (red) under different conditions. **a** Synthetic yeast cells expressing Cre_{UAG14} in the medium supplemented with varying concentration of OMeY (1, 2, 5 and 10 mM) by different ncAA concentration. **b** Synthetic yeast cells expressing Cre variants (Cre_{UAG5} and Cre_{UAG14}) in the medium supplemented with 1 mM OMeY. Source data are provided as a Source Data file.

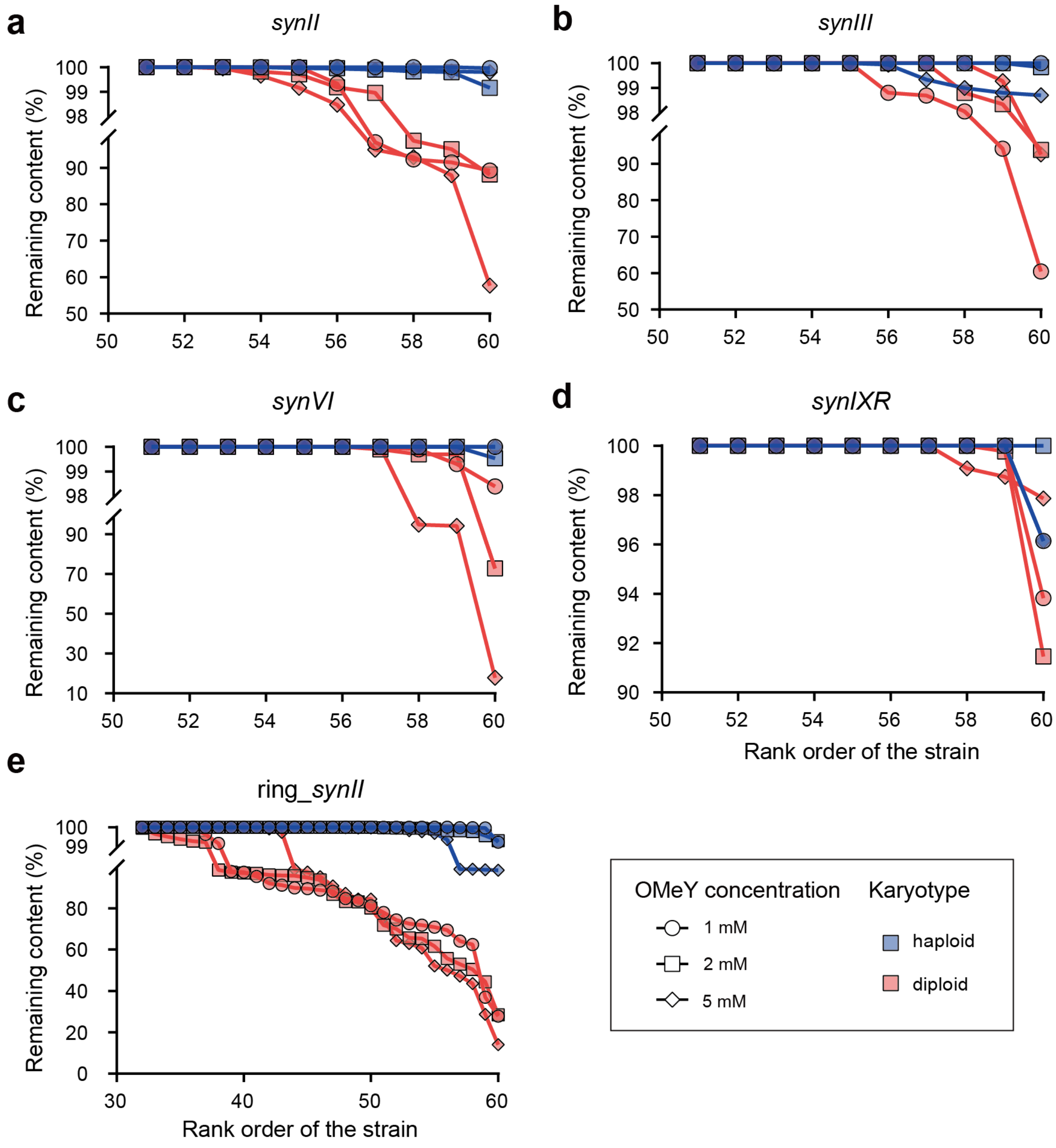

Supplementary Figure 4. Comparison of the remaining chromosome content between diploid and haploid strains that were subjected to GCE-SCRaMbLE under the same condition. Y axis represents the remaining content of each synthetic chromosome including *synII*, *synIII*, *synVI*, right arm of *synIX* (*synIXR*) and circular form of *synII* (ring_*synII*). Different synthetic chromosomes *synII* (**a**), *synIII* (**b**), *synVI* (**c**), *synIXR* (**d**) and ring_*synII* (**e**) were analyzed. X axis represents the number of strains in each group (total 60). Haploid and diploid strains are labeled as blue and red respectively. Circle, square and rhombus represent 1 mM, 2 mM and 5 mM OMeY concentration respectively. Source data are provided as a Source Data file.

Supplemental Table 1. Design of 23 groups of synthetic yeast cells for GCE-SCRaMbLE

Group	Synthetic chromosomes	Ploidy	Cre variants	OmeY concentration
1	*synII synIII synVI synIXR*	haploid	Cre_{UAG5}	1 mM
2	*synII synIII synVI synIXR*	haploid	Cre_{UAG14}	1 mM
3	*synII synIII synVI synIXR*	haploid	Cre_{UAG14}	2 mM
4	*synII synIII synVI synIXR*	haploid	Cre_{UAG14}	5 mM
5	*synII synIII synVI synIXR*	haploid	Cre_{UAG14}	10 mM
6	*synII*	haploid	Cre_{UAG5}	1 mM
7	*synII*	haploid	Cre_{UAG14}	1 mM
8	*synII*	haploid	Cre_{UAG14}	2 mM
9	*synII*	haploid	Cre_{UAG14}	5 mM
10	*synII*	haploid	Cre_{UAG14}	10 mM
11	ring_*synII*	haploid	Cre_{UAG5}	1 mM
12	ring_*synII*	haploid	Cre_{UAG14}	1 mM
13	ring_*synII*	haploid	Cre_{UAG14}	2 mM
14	ring_*synII*	haploid	Cre_{UAG14}	5 mM
15	ring_*synII*	haploid	Cre_{UAG14}	10 mM
16	*synII synIII synVI synIXR*	diploid	Cre_{UAG5}	1 mM
17	*synII synIII synVI synIXR*	diploid	Cre_{UAG14}	1 mM
18	*synII synIII synVI synIXR*	diploid	Cre_{UAG14}	2 mM
19	*synII synIII synVI synIXR*	diploid	Cre_{UAG14}	5 mM
20	ring_*synII*	diploid	Cre_{UAG5}	1 mM
21	ring_*synII*	diploid	Cre_{UAG14}	1 mM
22	ring_*synII*	diploid	Cre_{UAG14}	2 mM
23	ring_*synII*	diploid	Cre_{UAG14}	5 mM

Supplemental Table 2. List of strains and plasmids used in this study.

Strain, plasmid	Description	Source or reference
**Strains:**		
S. cerevisiae		
BY4741	*MATa* ura3Δ0 leu2Δ0 his3Δ1 met15Δ0	[1]
BY4742	*MATalpha* ura3Δ0 leu2Δ0 his3Δ1 lys2Δ0	[1]
synII	*MATa* ura3Δ0 leu2Δ0 his3Δ1 met15Δ0 synLYS2, *synII*	[2]
ring_*synII*	*MATa* ura3Δ0 leu2Δ0 his3Δ1 met15Δ0 synLYS2, ring_*synII*	This study
2369R	*MATalpha* ura3Δ0 leu2Δ0 his3Δ1 MET15 HO::SUP61 synLYS2, *synII*, *synIII*, *synVI*, *synIXR*	This study
ring_*synII* Dip	Diploid, ring_*synII* mating with BY4742	This study
2369R Dip	Diploid, 2369R mating with BY4741	This study
E. coli		
BL21(DE3)	F– *omp*T *hsdS$_B$* (r_B–, m_B–) *gal dcm* (DE3)	Vazyme Cat number: C504-02
**Plasmids:**		
pET28a	Km^r	MiaoLingPlasmid Cat number: P0023
pRS415	Ap^r; LEU2.Shuttle plasmid, empty vector	[3]
pRS413	Ap^r; HIS3.Shuttle plasmid, empty vector	[3]
pXF231	pRS415 LeuOmeRS pair	[4]
pXF220	pRS413 carries Cre_{UAG5}	This study
pXF221	pRS413 carries Cre_{UAG11}	This study
pXF238	pRS413 carries Cre_{UAG14}	This study
pXF239	pRS413 carries Cre_{UAG18}	This study
pSCW11-Cre-EBD	Cre fused to EBD and controlled by daughter cell-specific promoter SCW11	[5]
pLH_Scr18	Km^r; URA3. Carries terminator between LoxP before GFP	[6]
pLH_Scr19	Km^r; URA3. Carries GFP	[6]

Ap^r, ampicillin resistance; Km^r, kanamycin resistance.

Supplemental Table 3. List of primes used in this study.

**Primer Pair**	**Primer Sequence (5'-3')**	**Description**
Cre_F<br>Cre_R	GCCGCGCGGCAGCCATATGTCCAATTTACTGACCGTACAC<br>GTGCGGCCGCAAGCTTCAATCGCCATCTTCCAGCAG	To amplify Cre for cloning into pET28a vector
Cre_EBD_R	CGAGTGCGGCCGCAAGCTTCAGACTGTGGCAGGGAAAC	To amplify Cre-EBD for cloning into pET28a together with Cre_F
Vec_F<br>Vec_R	TGAAGCTTGCGGCCGCACTCGAGCACC<br>GTAAATTGGACATATGGCTGCCGCGCGGC	For inverse PCR to amplify pET28a backbone
Remove_EBD_F<br>Remove_EBD_R	GTAGAATGCCTGATATTGACTCAATATCCGTTGCG<br>GATATTGAGTCAATATCAGGCATTCTACTCAATCGCCATCTTCCAGCAGGACC	To remove EBD on pSCW11-Cre-EBD
Cre UAG5_F<br>Cre UAG5_R	TATCTGAGTGTTAAATGTCCAATTTATAGACCGTACACCAAAATTTGCCTGCAT<br>CTGTACACTTTACTTAAAACCATTATCTGAGTGTTAAATGTCCAATTTA	To create Cre variant Cre_{UAG5}
Cre UAG11_F<br>Cre UAG11_R	CCAATTTACTGACCGTACACCAAAATTAGCCTGCATTACCGGTCGATGCAAC<br>GTGTTAAATGTCCAATTTACTGACCGTACACCAAAAT	To create Cre variant Cre_{UAG11}
Cre UAG14_F<br>Cre UAG14_R	GACCGTACACCAAAATTTGCCTGCATAGCCGGTCGATGCAACGAGTGATGAGG<br>CCAATTTACTGACCGTACACCAAAATTTGCCTGCA	To create Cre variant Cre_{UAG14}
Cre UAG18_F<br>Cre UAG18_R	TTTGCCTGCATTACCGGTCGATTAGACGAGTGATGAGGTTCGCAAGAA<br>TTTGCCTGCATTACCGGTCGAT	To create Cre variant Cre_{UAG18}
MAT_F<br>MATα_R<br>MATa_R	AGTCACATCAAGATCGTTTATGG<br>GCACGGAATATGGGACTACTTCG<br>ACTCCACTTCAAGTAAGAGTTTG	Mating type verification

References

1 Brachmann, C. B. *et al.* Designer deletion strains derived from Saccharomyces cerevisiae S288C: a useful set of strains and plasmids for PCR-mediated gene disruption and other applications. *Yeast* **14**, 115-132 (1998).

2 Shen, Y. *et al.* Deep functional analysis of synII, a 770-kilobase synthetic yeast chromosome. *Science* **355** (2017).

3 Kitazono, A. A. Optimized protocols and plasmids for in vivo cloning in yeast. *Gene* **484**, 86-89 (2011).

4 Wu, N., Deiters, A., Cropp, T. A., King, D. & Schultz, P. G. A genetically encoded photocaged amino acid. *J Am Chem Soc* **126**, 14306-14307 (2004).

5 Dymond, J. S. *et al.* Synthetic chromosome arms function in yeast and generate phenotypic diversity by design. *Nature* **477**, 471-476 (2011).

6 Hochrein, L., Mitchell, L. A., Schulz, K., Messerschmidt, K. & Mueller-Roeber, B. L-SCRaMbLE as a tool for light-controlled Cre-mediated recombination in yeast. *Nat Commun* **9**, 1931 (2018).
