# Supplementary material for: Systematic dissection of key factors governing recombination outcomes by GCE-SCRaMbLE
Source: Nat Commun. 2022 Oct 3;13:5836. doi: 10.1038/s41467-022-33606-0 (PMC9530153; doi:10.1038/s41467-022-33606-0)
Supplement: Supplementary file 2 — Reporting Summary [file 41467_2022_33606_MOESM2_ESM.pdf]

nature portfolio

Corresponding author(s): Yue Shen & Xian Fu

Last updated by author(s): Sep 21, 2022

Reporting Summary

Nature Portfolio wishes to improve the reproducibility of the work that we publish. This form provides structure for consistency and transparency in reporting. For further information on Nature Portfolio policies, see our Editorial Policies and the Editorial Policy Checklist.

Statistics

For all statistical analyses, confirm that the following items are present in the figure legend, table legend, main text, or Methods section.

n/a	Confirmed	
☐	☒	The exact sample size (*n*) for each experimental group/condition, given as a discrete number and unit of measurement
☐	☒	A statement on whether measurements were taken from distinct samples or whether the same sample was measured repeatedly
☐	☒	The statistical test(s) used AND whether they are one- or two-sided<br>*Only common tests should be described solely by name; describe more complex techniques in the Methods section.*
☒	☐	A description of all covariates tested
☒	☐	A description of any assumptions or corrections, such as tests of normality and adjustment for multiple comparisons
☐	☒	A full description of the statistical parameters including central tendency (e.g. means) or other basic estimates (e.g. regression coefficient) AND variation (e.g. standard deviation) or associated estimates of uncertainty (e.g. confidence intervals)
☐	☒	For null hypothesis testing, the test statistic (e.g. *F*, *t*, *r*) with confidence intervals, effect sizes, degrees of freedom and *P* value noted<br>*Give P values as exact values whenever suitable.*
☒	☐	For Bayesian analysis, information on the choice of priors and Markov chain Monte Carlo settings
☒	☐	For hierarchical and complex designs, identification of the appropriate level for tests and full reporting of outcomes
☒	☐	Estimates of effect sizes (e.g. Cohen's *d*, Pearson's *r*), indicating how they were calculated

Our web collection on statistics for biologists contains articles on many of the points above.

Software and code

Policy information about availability of computer code

Data collection	No software was used for data collection.
Data analysis	Data analysis is described in method. The raw data was filtered by SOAPnuke (1.5.6). The filtered reads were mapped by SOAPaligner (2.21). The recombination sites were screened by Bowtie2 (2.3.5.1). The recombined chromosomes were reconstructed according to previously reported method (Shen, Y. et al. SCRaMbLE generates designed combinatorial stochastic diversity in synthetic chromosomes. Genome Res 26, 36-49 (2016).). Hi-C data was analyzed and normalized by HiC-Pro (3.1.0). The 3D coordinate values of contact maps were generated by ShRec3D (1.0) (https://github.com/jbmorlot/ShRec-Exented). The 3D chromosome was displayed by PyMOL (2.0). Quantitative signals of blots and gels were quantified by ImageJ (version 1.53e). Data is processed by Excel (Office 2019) and GraphPad Prism (9.1.0).

For manuscripts utilizing custom algorithms or software that are central to the research but not yet described in published literature, software must be made available to editors and reviewers. We strongly encourage code deposition in a community repository (e.g. GitHub). See the Nature Portfolio guidelines for submitting code & software for further information.

March 2021

Data

Policy information about availability of data

All manuscripts must include a data availability statement. This statement should provide the following information, where applicable:

- Accession codes, unique identifiers, or web links for publicly available datasets
- A description of any restrictions on data availability
- For clinical datasets or third party data, please ensure that the statement adheres to our policy

The DNA sequencing data of the SCRaMbLEd synthetic yeast strains that support the findings of this study have been deposited in the NCBI database under accession code PRJNA876960 and CNSA (CNGB Nucleotide Sequence Archive) under accession number CNP0002899 (https://db.cngb.org/search/project/CNP0002899/). The reference genome of BY4741 is downloaded from the Saccharomyces Genome Database (http://sgd-archive.yeastgenome.org/sequence/strains/BY4741/BY4741_Toronto_2012/). The reference sequence of synII, synIII, synVI, and synIXR used in this study can be downloaded from GenBank with accession code CP013608, KC880027, SRX2589074, and JN020955, respectively. All data supporting the findings of this study are available within the manuscript file and its Supplementary Information files. Source data are provided with this paper.

Human research participants

Policy information about studies involving human research participants and Sex and Gender in Research.

Reporting on sex and gender	N/A
Population characteristics	N/A
Recruitment	N/A
Ethics oversight	N/A

Note that full information on the approval of the study protocol must also be provided in the manuscript.

Field-specific reporting

Please select the one below that is the best fit for your research. If you are not sure, read the appropriate sections before making your selection.

☒ Life sciences ☐ Behavioural & social sciences ☐ Ecological, evolutionary & environmental sciences

For a reference copy of the document with all sections, see nature.com/documents/nr-reporting-summary-flat.pdf

Life sciences study design

All studies must disclose on these points even when the disclosure is negative.

Sample size	n=6 were chosen for fluorescent assays in Fig. 1B; we deem this number to be appropriate as convention in similar reports is n=3. After confirming the reproducibility of our fluorescent assays, biological replicates (n=3) were chosen for remaining similar experiments (Fig. 1C-D). Three biological replicates were used for recombination enzymatic assays and survival rate measurement, as this sample size is standard for biochemical assay and microbial growth assay.<br><br>For SCRaMbLEd genome reconstruction and rearrangement analysis, total 1380 (=23×60) isolated yeast strains subjected to GCE-SCRaMbLEd were selected for sequencing. N=23 represents 23 test groups that were designed to test different factors affecting SCRaMbLE process, and N=23 is enough to cover all different factors and is a reasonable sample size considering the cost, time and efforts of sequencing and whole-genome reconstruction of SCRaMbLEd yeast strains. We selected 60 replicates in each group based on the SCRaMbLE rate (30%) and sequencing cost; in addition, this sample size is close to a previous similar study (N=64, Genome Res. 2016 Jan;26(1):36-49).
Data exclusions	All data are included for analysis.
Replication	All assays including fluorescence assay, western blot, and enzymatic experiments were repeated 3-6 times independently as indicated in the figure legends and showed similar results.
Randomization	Replicates were randomized across micro-plate fluorescent assays to avoid position effects. When picking up 1380 clones after GCE-SCRaMbLE for whole genome sequencing, all clones were picked at random from agar plates. Clones with different colony size and growing in different areas on agar plate were all randomly picked up to avoid position effects and phenotypic effects
Blinding	Blinding is not relevant because no group allocation was involved in this study.

Reporting for specific materials, systems and methods

We require information from authors about some types of materials, experimental systems and methods used in many studies. Here, indicate whether each material, system or method listed is relevant to your study. If you are not sure if a list item applies to your research, read the appropriate section before selecting a response.

Materials & experimental systems

n/a	Involved in the study
☐	☒ Antibodies
☒	☐ Eukaryotic cell lines
☒	☐ Palaeontology and archaeology
☒	☐ Animals and other organisms
☒	☐ Clinical data
☒	☐ Dual use research of concern

Methods

n/a	Involved in the study
☒	☐ ChIP-seq
☒	☐ Flow cytometry
☒	☐ MRI-based neuroimaging

Antibodies

Antibodies used	Cre-specific monoclonal antibody (1:1000) (Invitrogen Cat# MA5-27870)<br><br>Alkaline phosphatase-linked anti-mouse IgG peroxidase antibody (1:1000) (Sigma-aldrich, Cat #: A2304)
Validation	All antibodies were used in the study according to the profile of manufacturers. Antibody validation was validated by the supplier as demonstrated in manufacturer's website and confirmed in this study to perform Western-Blot in Fig. 3a.<br><br>Validation of Cre-specific monoclonal antibody according to the manufacturer's website (https://www.thermofisher.cn/cn/zh/antibody/product/Cre-recombinase-Antibody-clone-GT10212-Monoclonal/MA5-27870)<br><br>Validation Alkaline phosphatase-linked anti-mouse IgG peroxidase antibody according to the manufacturer's website (https://www.sigmaaldrich.cn/CN/zh/product/sigma/a2304)
